# Vanillic acid from *Actinidia deliciosa* impedes virulence in *Serratia marcescens* by affecting S-layer, flagellin and fatty acid biosynthesis proteins

**DOI:** 10.1038/s41598-017-16507-x

**Published:** 2017-11-27

**Authors:** Sivasamy Sethupathy, Sivagnanam Ananthi, Anthonymuthu Selvaraj, Balakrishnan Shanmuganathan, Loganathan Vigneshwari, Krishnaswamy Balamurugan, Sundarasamy Mahalingam, Shunmugiah Karutha Pandian

**Affiliations:** 1 Department of Biotechnology, Alagappa University, Science Campus, Karaikudi, 630 003 Tamil Nadu India; 2 Laboratory of Molecular Virology and Cell Biology, Department of Biotechnology, Indian Institute of Technology Madras, Chennai, 600 036 Tamil Nadu India

## Abstract

*Serratia marcescens* is one of the important nosocomial pathogens which rely on quorum sensing (QS) to regulate the production of biofilm and several virulence factors. Hence, blocking of QS has become a promising approach to quench the virulence of *S. marcescens*. For the first time, QS inhibitory (QSI) and antibiofilm potential of *Actinidia deliciosa* have been explored against *S. marcescens* clinical isolate (CI). *A. deliciosa* pulp extract significantly inhibited the virulence and biofilm production without any deleterious effect on the growth. Vanillic acid was identified as an active lead responsible for the QSI activity. Addition of vanillic acid to the growth medium significantly affected the QS regulated production of biofilm and virulence factors in a concentration dependent mode in *S. marcescens* CI, ATCC 14756 and MG1. Furthermore vanillic acid increased the survival of *Caenorhabditis elegans* upon *S. marcescens* infection. Proteomic analysis and mass spectrometric identification of differentially expressed proteins revealed the ability of vanillic acid to modulate the expression of proteins involved in S-layers, histidine, flagellin and fatty acid production. QSI potential of the vanillic acid observed in the current study paves the way for exploring it as a potential therapeutic candidate to treat *S. marcescens* infections.

## Introduction

*Serratia marcescens is* an opportunistic human pathogen responsible for severe nosocomial infections^1^. Though *S. marcescens* was previously viewed as an innocuous organism, later on, it has been reported as an etiological agent of hospital-associated infections including septicemia, meningitis, endocarditis, urinary tract, respiratory tract, bloodstream and wound infections^2^. Prevalence of *S. marcescens* in healthcare settings is increasing parallel with the emergence of multi-drug resistance^3,4^. Biofilm formation is one of the primary underlying mechanisms responsible for antibiotic resistance in *S. marcescens*. This adherent nature of *S. marcescens* utilizes living as well as non-living surfaces such as biomedical devices to form the biofilm, finally leads to life-threatening infections^5^. A recent report states that biofilms produced by *S. marcescens* clinical isolates require supratherapeutic doses of kanamycin, gentamicin and chloramphenicol as minimum inhibitory concentration, which is 10, 100 and 1000 times higher concentration respectively, to kill the planktonic cells^6^.

Pathogenicity and biofilm formation of *S. marcescens* is directed by the cell density mediated gene expression mechanism called quorum sensing (QS). Acyl-homoserine lactones (AHLs) are the well-known autoinducers in LuxI/R QS system of Gram-negative bacteria^7^. Strains of *S. marcescens* have been reported to utilize a wide range of AHL molecules to regulate the transcription of target genes involved in biofilm formation, swimming and swarming motility, production of red coloured pigment prodigiosin, extracellular virulence enzymes (lipase, protease and nuclease), hemolysin and surfactants^7^. Briefly, the most common QS signal molecule used by the genus *Serratia* is N-hexanoyl-L-homoserine lactone (C6-HSL). The *Serratia* spp. strain ATCC 39006^8^, *S. marcescens* MG1^9^ and *S. marcescens* strain 12 utilizes N-butanoyl homoserine lactone (C4-HSL)^10^ as a QS signal molecule. In addition to C6-HSL, *S. marcescens* SS-1 also produces N-(3-oxohexanoyl) homoserine lactone (3-Oxo-C6-HSL), N-heptanoyl homoserine lactone (C7-HSL) and N-octanoyl homoserine lactone (C8-HSL) to regulate its QS dependent virulence factor production^8^. Hence, quenching its QS through the bioactive compounds from natural sources is a promising alternative strategy to combat infections caused by *S. marcescens*. For example, compounds such as O-methyl ellagic acid^11^ and alpha-bisabolol^12^, inhibiting QS in *S. marcescens* have been known to reduce the protease, lipase, prodigiosin and biofilm formation.

In recent years, awareness of the consumption of fruits for health promotion has increased. *Actinidia deliciosa* (Kiwifruit) has been classified as an excellent source of vitamin C, dietary fibre, vitamin E and potassium, based on the US FDA’s definition^13^. Kiwifruit has been known for its antioxidant, cardiovascular preventive and laxative activities^14^. Furthermore, consumption of kiwifruit selectively enhances the growth of intestinal bacteria such as Bifidobacterium and Lactobacillus^15^. Since the QSI potential of kiwifruit has never been explored and the presence of a wide range of secondary metabolites in kiwifruit has eventually led our focus to explore its QSI potential against *S. marcescens*.

## Results and Discussion

### Evaluation of QSI potential of *A. deliciosa* pulp extract (ADPE) against *S. marcescens* and identification of active principle

**Figure 1:**
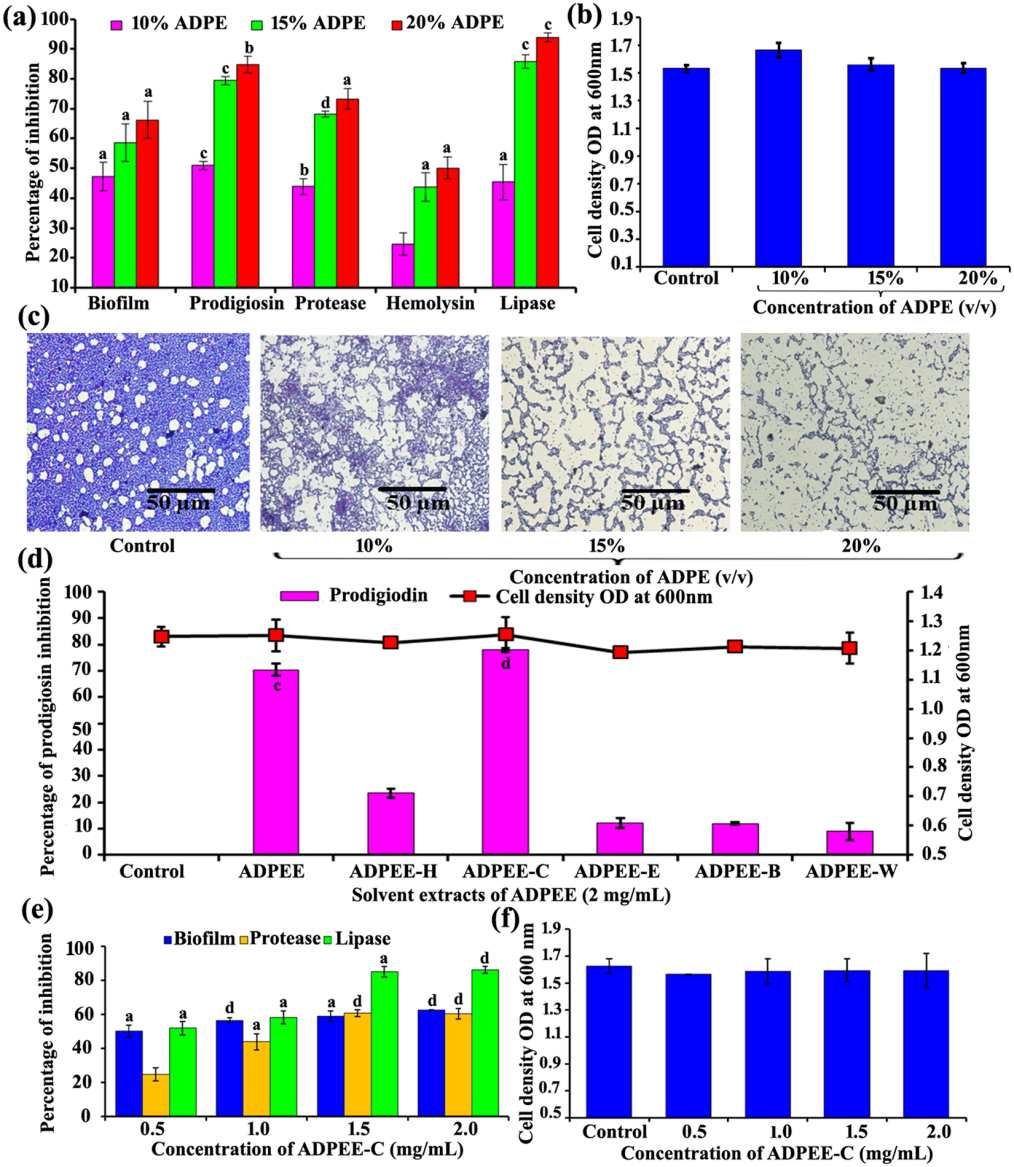
Quorum sensing inhibitory potential of ADPE against *S. marcescens*. The ADPE inhibited the QS regulated virulence factors such as biofilm formation, prodigiosin, protease, hemolysin and lipase (**a**) in a dose dependent manner without inhibiting the growth of *S. marcescens* (**b**). Light microscopic visualization of *S. marcescens* biofilm formed in the absence and presence of ADPE (**c**). Effect of solvent extracts of ADPEE against prodigiosin production and the growth (**d**) of *S*. *marcescens*. ADPEE-C exhibited the concentration dependent inhibition of biofilm formation, protease and lipase (**e**) without affecting the growth (**f**). Growth of *S. marcescens* was measured at 600 nm after 18 h incubation at 30 °C. Error bars represent standard deviations from the mean (n = 6 [biological triplicates in experimental duplicates]). Statistical significance was analyzed using one way ANOVA-Duncan’s *post-hoc* test. ^a, b, c^ and ^d^ indicate the significant difference p < 0.05, p < 0.01, p < 0.005 and p < 0.001, respectively.

A number of studies have shown that bacteria, fungi and plants produce QSI compounds to protect themselves and compete with invading organisms. Due to their non-toxic and multifunctional nature, QSIs from edible sources are gaining attention in augmenting antimicrobial therapy^16^. *S. marcescens* strains from environmental and clinical origins were able to produce the intracellular prodigiosin pigment. Since prodigiosin production is under the direct regulation of the QS signalling mechanism^17^; the QSI potential of ADPE was initially assessed by prodigiosin assay. ADPE exhibited concentration-dependent prodigiosin inhibitory activity (Fig. 1a). At a higher concentration (20%) ADPE greatly reduced the prodigiosin production (85%) in *S. marcescens* when compared to control (Fig. 1a). The prodigiosin inhibitory activity of ADPE clearly suggests the presence of QS inhibitor(s).

In addition, dose-dependent inhibition of QS-regulated production of haemolysin (Fig. 1a) and biofilm formation (Fig. 1a) was observed with ADPE treatment without any reduction in the growth of *marcescens* (Fig. 1b). Light microscopic observation of biofilms formed in the presence and absence of ADPE further confirmed the potent antibiofilm activity (Fig. 1c). These results affirm the ability of ADPE to interfere with the QS mechanism of *S. marcescens*.

To identify the active lead(s) responsible for the QSI activity of ADPE, the ethanolic extract of ADPE (ADPEE) was extracted subsequently with hexane (ADPEE-H), chloroform (ADPEE-C), ethyl acetate (ADPEE-E) and butanol (ADPEE-B). The QSI activity of the dried solvent extracts was assessed based on its ability to reduce the QS-regulated prodigiosin production. The highest percentage of prodigiosin pigment reduction was observed in the ADPEE-C extract at 2 mg/mL (Fig. 1d). Henceforth, the effect of ADPEE-C extract on biofilm formation, production of protease and lipase production of *S. marcescens* was analysed. The ADPEE-C extract also showed a concentration-dependent inhibition of biofilm, protease and lipase (Fig. 1e) without any interference in the growth of *S. marcescens* (Fig. 1f).

The major constituents of ADPEE-C extract were identified using GC-MS analysis. The major components found in the ADPEE-C extract are vanillic acid (34.43%) and n-Hexadecanoic acid (11.85%) (Supplementary Fig. 1). Other major constituents present in the ADPEE- C are listed in supplementary Table 1. Vanillic acid is a well-known generally regarded as safe (GRAS) flavoring agent with antioxidant, anti-lipid peroxidative, anti-inflammatory and neuroprotective/cognitive effects.

### Vanillic acid inhibits the QS regulated virulence factors and biofilm formation in S*. marcescens*

**Figure 2:**
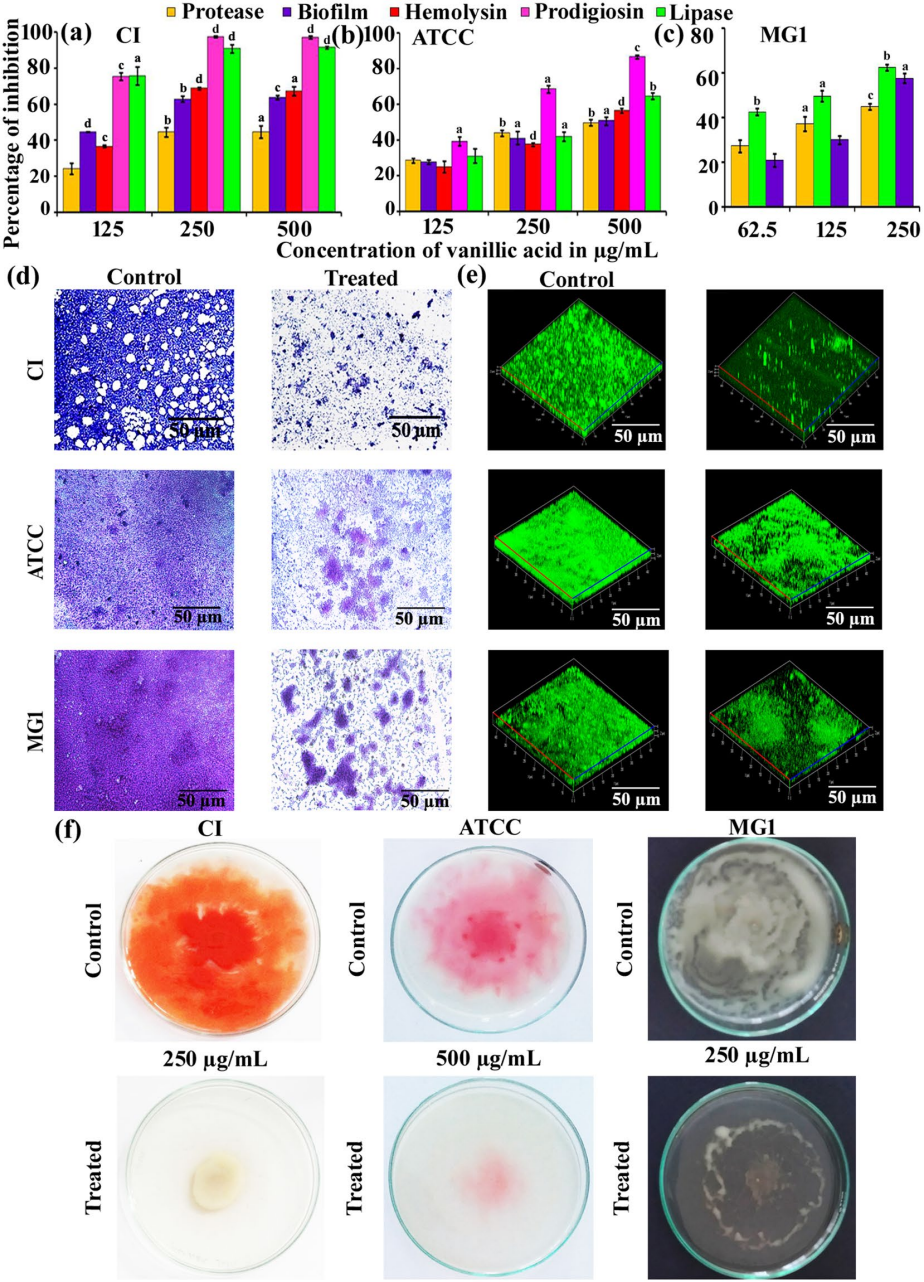
Quorum sensing inhibitory potential of active lead vanillic acid. Effect of pure vanillic acid on the QS regulated biofilm formation and prodigiosin, protease, hemolysin and lipase production of *S. marcescens* CI, *S. marcescens* ATCC and *S. marcescens* MG1 (**a**). Light (**b**) and CLSM [Three dimensional micrographs] (**c**) analyses corroborate the antibiofilm activity of vanillic acid. Effect of vanillic acid on the swarming motility of *S. marcescens* CI, *S. marcescens* ATCC and *S. marcescens* MG1 (**d**). Error bars represent standard deviations from the mean (n = 6 [biological triplicates in experimental duplicates]). Statistical significance was analyzed using one way ANOVA-Duncan’s *post-hoc* test. ^a, b, c^ and ^d^ indicate the significant difference p < 0.05, p < 0.01, p < 0.005 and p < 0.001, respectively.

Pure vanillic acid was assayed for its QSI activity against *S. marcescens* CI, *S. marcescens* ATCC and *S. marcescens* MG1 virulence factors. Extracellular proteases of *S. marcescens* modulate the host’s immune response by inducing the inflammatory response^18^. Compounds/extracts inhibiting the production of protease can be used to potentiate the host’s innate immune response as well as antimicrobial therapy. In this context, the concentration-dependent decrease in the production of the secreted protease was observed in the cell free culture supernatant (CFCS) of *S. marcescens* CI (Fig. 2a), *S. marcescens* ATCC 14756 (Fig. 2b) and *S. marcescens* MG1 (Fig. 2c) grown in the presence of vanillic acid.

Lipolytic enzymes are utilised by most of the bacterial pathogens to establish the infection by degrading the phospholipid bilayer of host cells and to manipulate the host cell signalling pathways^19^. Production of lipase was significantly reduced in all the three *S. marcescens* used in the present study. Briefly, 94 and 62% of inhibition of lipase production was noted in *S. marcescens* CI (Fig. 2a) and *S. marcescens* MG1 (Fig. 2b) respectively, at 250 µg/mL of vanillic acid. Whereas at 500 µg/mL of vanillic acid 65% of lipase inhibition was observed in *S. marcescens* ATCC 14756 (Fig. 2c). Haemolysin is a well-characterised virulence factor of *S. marcescens*. Vanillic acid reduced the haemolytic activity of *S. marcescens* CI and *S. marcescens* ATCC in a concentration-dependent mode. At 250 and 500 µg/mL of vanillic acid 68.75 and 56.52% of haemolysin production was observed in *S. marcescens* CI (Fig. 2a) and *S. marcescens* ATCC (Fig. 2b), respectively. These results show that vanillic acid has a potential to interfere with the QS mechanism of *S. marcescens*.

Biofilm formation has been considered as a major event in the establishment of chronic infections and confers extreme resistance to a broad spectrum of antibiotics. Vanillic acid has been reported for its ability to inhibit QS-dependent violacein pigment production in *C. violaceum* ^20^ and biofilm formation in *Aeromonas hydrophila* ^21^. Since QS is involved in the biofilm formation of *S. marcescens* ^22^, the anti-QS activity of vanillic acid is expected to have a significant negative effect on biofilm formation as well as swarming motility. In the present study, vanillic acid exhibited dose-dependent antibiofilm activity against *S. marcescens* CI (Fig. 2a), *S. marcescens* ATCC (Fig. 2b) and *S. marcescens* MG1 (Fig. 2c). Briefly, 63.6 and 57.64% of biofilm inhibition was noted at 250 µg/mL of vanillic acid in *S. marcescens* CI (Fig. 2a) and *S. marcescens* MG1 (Fig. 2c), respectively. At the same time, 50.84% (Fig. 2a) of biofilm inhibition was observed in *S. marcescens* ATCC grown in the presence of 500 µg/mL of vanillic acid.

In addition, biofilms of *S. marcescens* CI, *S. marcescens* ATCC and *S. marcescens* MG1 formed in the absence and presence of vanillic acid were observed under light microscope and the results revealed the reduction in biofilm formation and surface coverage (Fig. 2d). CLSM analysis also further confirmed the antibiofilm potential of vanillic acid (Fig. 2e). Swarming motility confers adaptive resistance to antibiotics and allows pathogenic bacteria to move across the moist, solid and viscous medium. Presence of vanillic acid in the growth medium was found to inhibit the swarming motility of all the three *S. marcescens* strains used in the present study (Fig. 2f).

**Figure 3:**
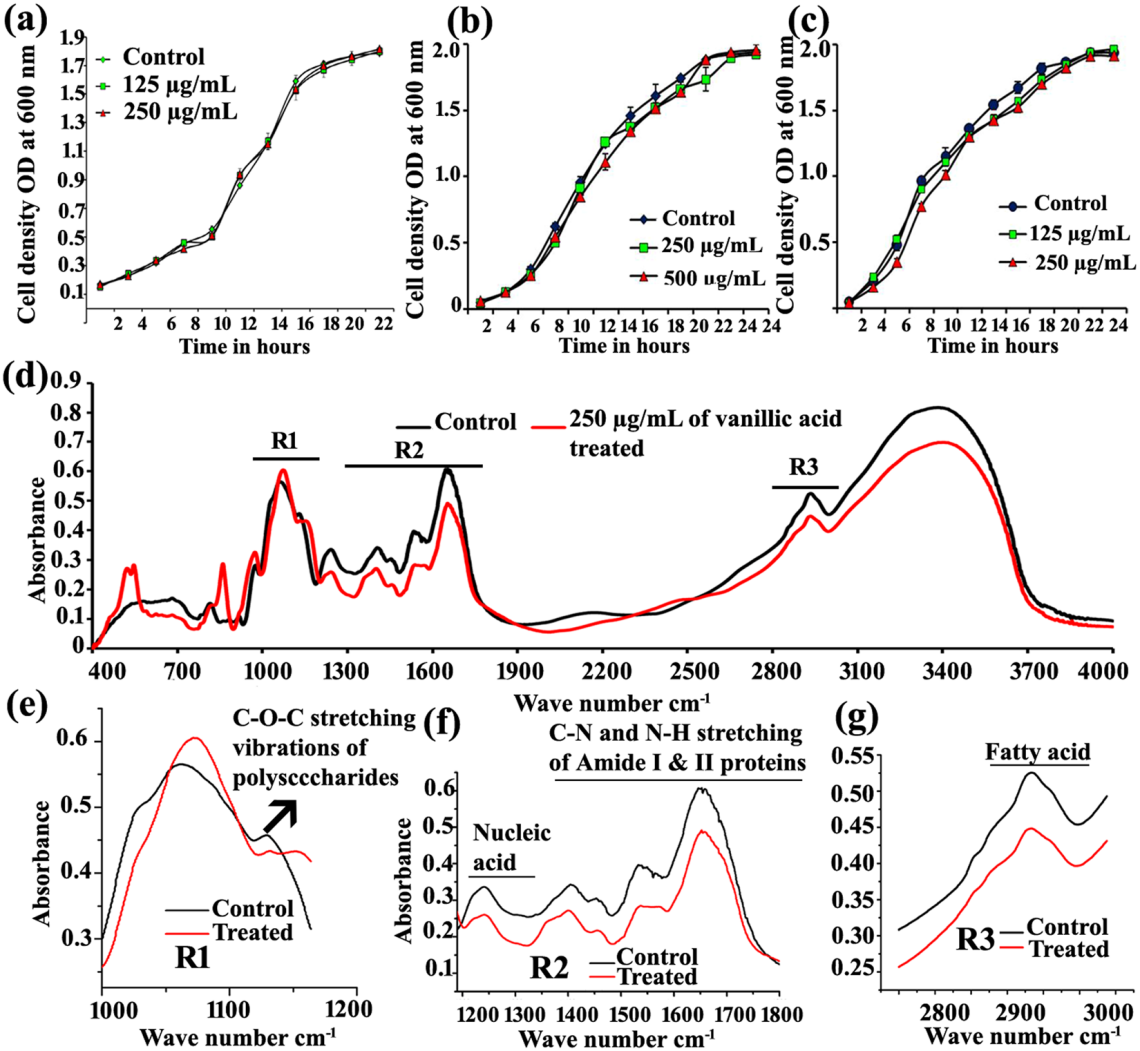
Growth curve analysis depicting the non-antibacterial/bacteriostatic nature of *S. marcescens* CI (**a**), *S. marcescens* ATCC (**b**) and *S. marcescens* MG1 (**c**) grown in the presence and absence of vanillic acid. Error bars represent standard deviations from the mean (n = 3 [biological triplicates]). FTIR analysis of EPS extracted from control and vanillic acid treated *S. marcescens* CI (**d**). Panel e, f and g depict the variations in the polysaccharide, nucleic acid, proteins and fatty acid region of EPS, respectively.

As an ideal QSI is expected to have no/least interference with the basal growth of bacteria^23^, the effect of vanillic acid on the growth of *S. marcescens* CI, *S. marcescens* ATCC and *S. marcescens* MG1 was assessed by measuring the cell density at 600 nm. Results of growth measurement clearly suggest that vanillic acid does not have antibacterial activity at the tested concentrations. In addition, growth curve analysis was also carried out and the results revealed the non-antibacterial/bacteriostatic nature of vanillic acid at 125, 250 and 500 µg/mL (Fig. 3a,b and c).

FT-IR analysis showed the presence of polysaccharides, nucleic acid, proteins and fatty acids in extracted EPS of *S. marcescens* control and vanillic acid treated (Fig. 3c). The presence of peaks at 1,200–900 cm^−1^ indicates the C–OH stretching and C–O–C, C–O ring vibrations of carbohydrates^24,25^ peaks at 1,250–1,220 cm^−1^ corresponds to the phosphodiester, free phosphate, and monoester phosphate functional groups of phosphodiester, DNA/RNA backbone, phospholipids and phospho sugars^26^. Peaks at 1,540 and 1,650 cm^−1^ indicated the N–H bending, C–N stretching of Amide II and C = O stretching, C–N bending of Amide I proteins, respectively^25^ (Fig. 3c). In addition, a peak at 3,000–2,800 cm^−1^ corresponds to C–H vibrations of the functional groups of fatty acids^24,25^. The polysaccharide region (1,200–900 cm^−1^) of vanillic acid treated EPS was found to be increased slightly. However reduction in the absorbance of a peak at 1120 cm^−1^–1140 cm^−1^ which corresponds to the C-O-C stretching vibrations of carbohydrates was observed in vanillic acid treated EPS. Whereas the decrease in the IR absorbance at 1,250–1,220 cm^−1^(nucleic acid)^27,28^, 1,540 and 1,650 cm^−1^(Amide II and Amide I proteins)^26^, 3,000–2,800 cm^−1^ (fatty acid)^25,29^ was observed in vanillic acid treated EPS. Hence alterations in the biofilm architecture upon vanillic acid treatment could be attributed to the reduction of nucleic acid, amide I and II proteins and fatty acid content of EPS. When compared to the control, vanillic acid treated EPS showed a decrease in the absorbance of a broad peak at 3,800–3,100 cm^−1^ corresponding to –OH group (responsible for the hydration of EPS). Comparison of FT-IR spectra of control and treated EPS revealed a noticeable decrease in the IR absorbance of the nucleic acid, protein and fatty acid content and not in the carbohydrate content of the EPS (Fig. 3c). In addition to carbohydrate, other macromolecules such as nucleic acid, protein and fatty acid are also the main constituents required for the formation of EPS matrix. The decrease in the nucleic acid, protein and fatty acid content of EPS upon vanillic acid treatment could be one of the underlying mechanisms of its antibiofilm activity. Furthermore, the EPS inhibitory potential of vanillic acid is expected to decrease the survival of *S. marcescens* under *in vivo* conditions.

### *In vivo* anti-QS and antibiofilm activity of vanillic acid

**Figure 4:**
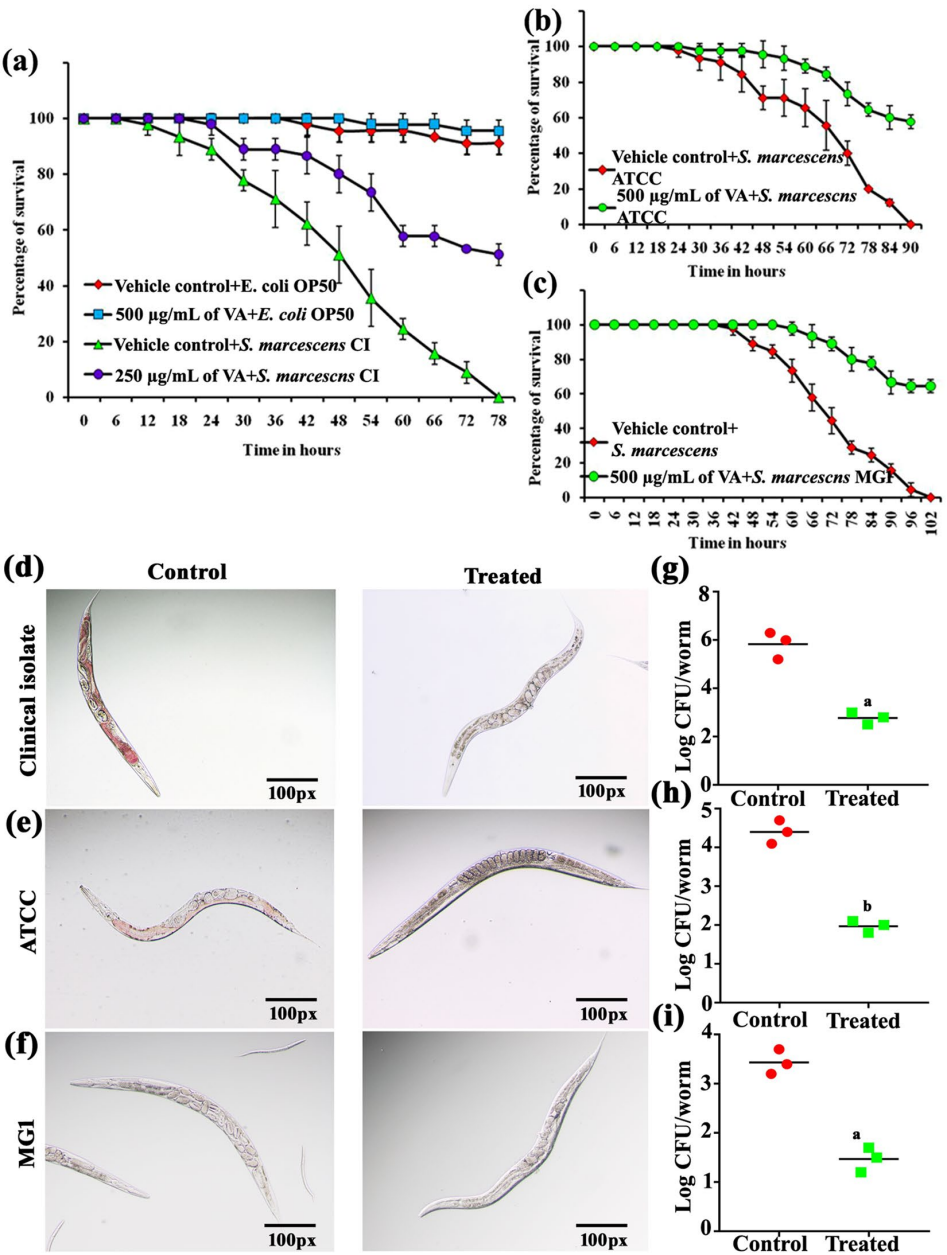
Survival graph showing the ability of vanillic acid treatment to rescue *C. elegans* from *S. marcescens* CI (**a**), *S. marcescens* ATCC (**b**) and *S. marcescens* MG1 (**c**) infection. Light micrographs depict the inhibition of prodigiosin pigment (**d** & **e**) and intestinal colonization (**d**,**e** & **f**) of *S. marcescens* upon vanillic acid treatment when compared to control. CFU assay results further confirm the inhibition of intestinal colonization of *S. marcescens* strains (**g**,**h** & **i**). Error bars represent standard deviations from the mean (n = 3 [biological triplicates in experimental duplicates]). Statistical significance was analyzed using one way ANOVA-Duncan’s *post-hoc* test. ^a^ and ^b^ indicate the significant difference p < 0.05 and p < 0.01, respectively.

*C. elegans* has been used as one of the simple and successful *in vivo* preclinical models to study the bacterial pathogenesis and screening of bioactive compounds with antibacterial, anti-QS and antibiofilm activities etc^30^. Anti-QS and antibiofilm agents have been already reported to rescue/increase the survival of *C. elegans* during pathogenic infection^31,32^. Hence, in the present study the *in vivo* anti-QS and antibiofilm activity of vanillic acid against *S. marcescens* CI, *S. marcescens* ATCC and *S. marcescens* MG1 are evaluates using *C. elegans*. Worm liquid-killing assay was used to evaluate the ability of the vanillic acid to rescue the *C. elegans* from *S. marcescens* infection. No significant difference in the survival of worms fed with *E. coli* OP50 + vehicle control and *E. coli* OP50 + 500 µg/mL of vanillic acid confirms the non-toxic nature of vanillic acid (Fig. 4a). In addition, results of killing assay revealed that *S. marcescens* CI (Fig. 4a) is more pathogenic to *C. elegans* than *S. marcescens* ATCC (Fig. 4b) and *S. marcescens* MG1 (Fig. 4c). Briefly, the complete killing (100%) was observed at 78 h in *S. marcescens* CI infected *C. elegans* (Fig. 4a). Whereas complete killing of *C*. *elegans* was observed at 90 h and 102 h upon *S. marcescens* ATCC (Fig. 4b) and *S. marcescens* MG1 (Fig. 4c), respectively. Interestingly, vanillic acid increases the survival of *C. elegans* during *S. marcescens* CI, *S. marcescens* ATCC and *S. marcescens* MG1 infection by 51 (Fig. 4a), 58 (Fig. 4b) and 64% (Fig. 4c), respectively. Inhibition of *in vivo* colonization and prodigiosin production was further confirmed using light microscopic analysis. From the light micrographs, it is clear that in the presence of vanillic acid *S. marcescens* CI (Fig. 4d) and *S. marcescens* ATCC (Fig. 4e) failed to produce prodigiosin and reduced intestinal colonization in *C. elegans*. Briefly, pink coloration/colonization was observed in the intestinal region of *C. elegans* upon *S. marcescens* CI (Fig. 4d) and *S. marcescens* ATCC (Fig. 4e) infection. Whereas no such coloration was noted in *C. elegans* in the presence of vanillic acid + *S. marcescens* CI (Fig. 4d) and vanillic acid + *S. marcescens* ATCC (Fig. 4e). In addition, *C. elegans* infected with *S. marcescens* CI (Fig. 4d), *S. marcescens* ATCC (Fig. 4e) and *S. marcescens* MG1 (Fig. 4f) exhibited damaged pharynx and internal hatching. Conversely, *C. elegans* infected with vanillic acid + *S. marcescens* strains showed typical pharynx and well-organized eggs in their uterus region (Fig. 4d,e and f). For further confirmation of the ability of the vanillic acid to inhibit intestinal colonization of *S. marcescens* CFU assay was carried out. Results showed a significant decrease in intestinal colonization of *S. marcescens* strains in the presence of vanillic acid when compared to control (Fig. 4g,h and i). From these observations, it is apparent that vanillic acid increases the survival of *C. elegans* by targeting biofilm formation and QS in *S. marcescens*.

### Effect of vanillic acid treatment on the cellular proteome of *S. marcescens*

**Figure 5:**
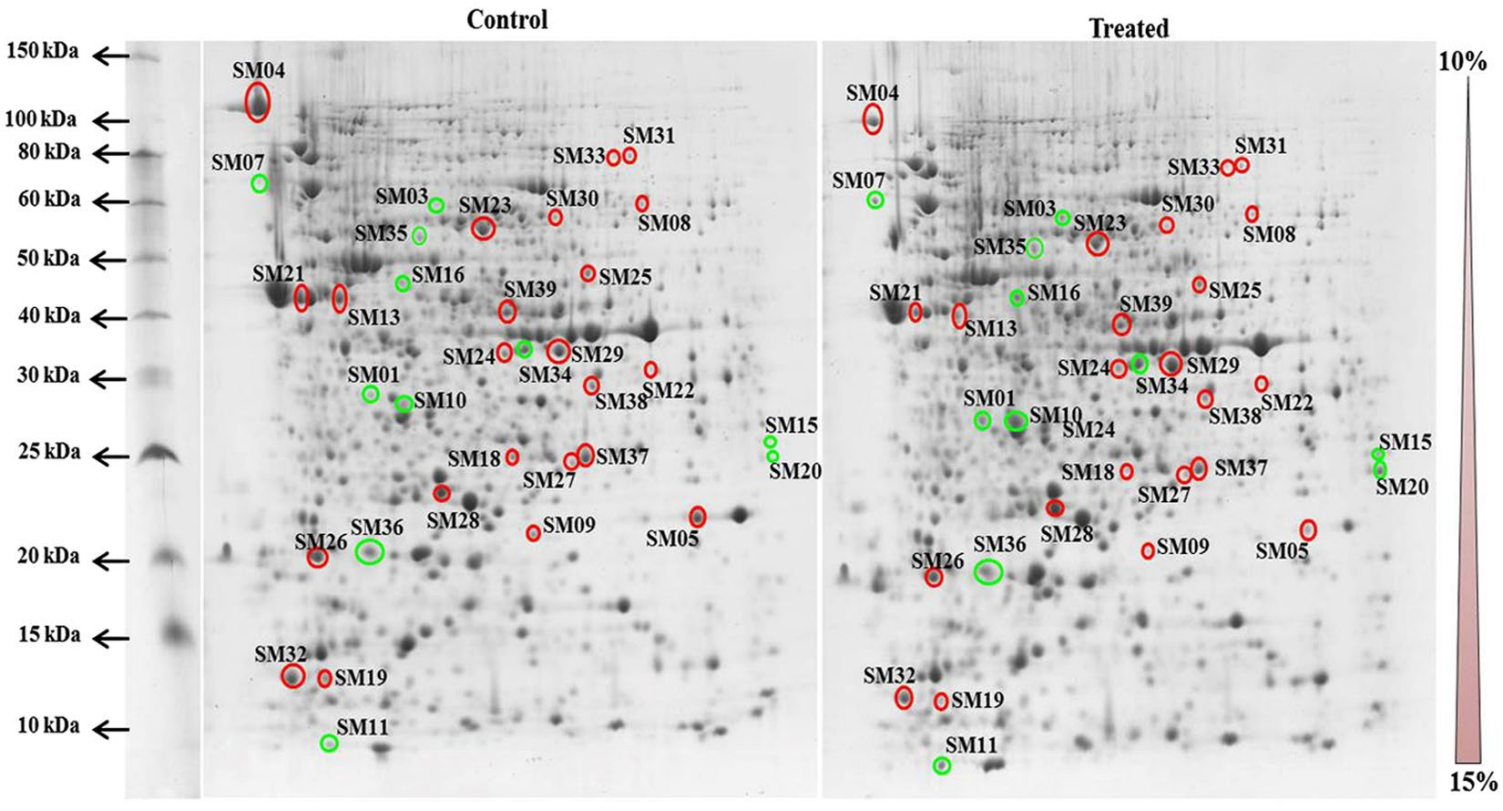
Representative gel pictures depicting the analysis of intracellular protein extract of *S. marcescens* CI grown in the absence and presence of 250 µg/mL of vanillic acid using 2-D gel electrophoresis. Each 450 µg of protein extract from control and treated cells were subjected to isoelectric focusing and resolved based on molecular weight in 10–15% gradient SDS-PAGE and protein spots were stained with MS compatible colloidal CBB. Up regulated and down regulated spots are encircled with green and red, respectively.

**Figure 6:**
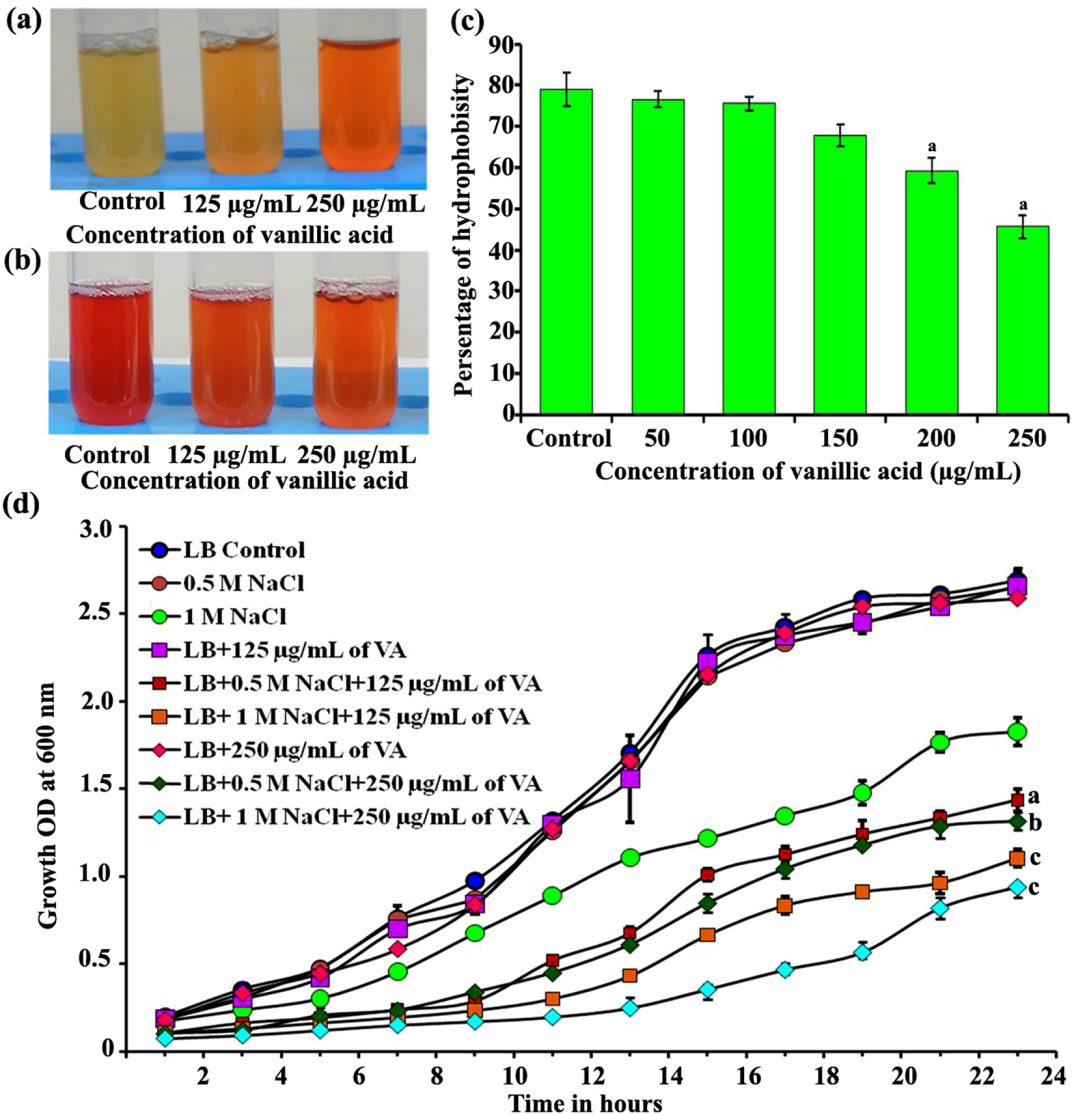
Gene ontology analysis of down and upregulated proteins of *S*. *marcescens* upon vanillic acid treatment.

The results of the virulence assays prompted us to study the underlying molecular mechanism of QS inhibition of vanillic acid in *S. marcescens*. To get an overview, cellular proteins (each 30 µg) from the exponential phase cultures of *S. marcescens* CI grown in the presence and absence of vanillic acid (250 μg/mL) were separated in SDS-PAGE. CBB stained SDS-PAGE gel showed interesting protein profile variations in the cellular proteome of *S. marcescens* cultured in the presence of vanillic acid when compared to the control. Then, the cellular proteome of control and vanillic acid treated *S. marcescens* CI were analysed using 2DGE in biological triplicates (Supplementary Fig. 2). The protein spots present in the control and treated gels were detected and matched using Image Master Platinum 7 software (GE Healthcare). Based on the densitometric analysis, among the detected spots (579), 27 spots and 21 spots were found to be down-regulated and upregulated by more than 1.5 fold, respectively (Fig. 5). Based on statistical significance (ANOVA 0.05), 19 down-regulated and 14 upregulated protein spots were selected for protein identification. Protein spots with more than 2 fold differential expression were identified using nano-LC MS/MS analysis. Furthermore, 1.9 to 1.5 fold differentially expressed protein spots were identified using MALDI-TOF/TOF analysis. The list of differentially expressed proteins and their functions are presented in Table 1. Gene ontology analysis of differentially expressed proteins revealed their involvement in the biosynthesis of flagella, amino acids, prodigiosin, outer membrane, carbohydrates and lipids of *S. marcescens* (Fig. 6).

**Table 1:** List of differentially expressed proteins of *S. marcescens* upon vanillic acid treatment identified using nano-LC-MS/MS and MALDI-TOF/TOF.

List of differentially expressed proteins of *S. marcescens* upon vanillic acid treatment identified using nano-LC-MS/MS analysis											
Spot No	Fold	ANOVA	Protein Score	Coverage (%)		No of peptides matched		Accession No			Description
SM1	5.77↑	0.005	820	75		6		A0A0G8BG56			Ferrous iron transporter B
SM3	4.74↑	0.012	1250	58		1		A0A0U7MA95			Aldehyde dehydrogenase B (aldB_1)
SM4	4.68↓	0.048	1514	13		3		A0A0N2A6Q2			Surface layer protein (slaA)
SM5	3.66↓	0.000	1172	39		1		A0A0U6CCF3			Uncharacterized protein
SM7	3.32↑	0.078	222	81		34		A0A086FJE2			60KDa chaperonin (groL)
SM8	2.72↓	0.034	533	69		34		A0A0G8B7Z6			Urocanate hydratase (hutU)
SM9	3.20↓	0.004	1486	78		24		A0A0P0QAE1			Molecular chaperone (OsmY)
SM10	3.01↑	0.001	3094	90		2		H9XP47			2,3- butanediol dehydrogenase (budC)
SM11	2.84↑	0.000	140	70		2		Q8GHK9			Multiple stress resistant protein (bhSA)
SM13	2.70↓	0.001	694	78		1		A0A0N2A3T4			Putative oxidoreductase
SM14	2.59↓	0.001	6159	94		19		A0A0N7JPV5			Flagellin
SM15	2.57↑	0.010	297	77		2		A0A0P0QGJ0			Long polar fibrial chaperon (lpfB)
SM16	2.40↑	0.001	1408	76		14		A0A0P0Q9Q2			Delta-aminolevulinic acid dehydratase
SM18	2.28↓	0.002	2660	85		2		A0A086FFY1			3-Oxoacyl-[acyl-carrier-protein] reductase (fabG)
SM20	2.17↑	0.079	1071	77		1		A0A0M4R8J3			Probable phospholipid-binding protein (mlaC)
SM21	2.11↓	0.003	5305	90		17		A0A0N7JPV5			Flagellin
SM22	2.00↓	0.042	1914	78		3		A0A0G8BBS9			Amidohydrolase
**List of differentially expressed proteins of** ***S. marcescens*** **upon vanillic acid treatment identified using MALDI-TOF/TOF**											
**Spot No**	**Fold**	**ANOVA**	**MOWSE Score**		**Coverage (%)**		**No of peptides matched**		**Accession No**	**Description**	
SM23	1.91↓	0.05	15118		9.3		7		Q5W264	4hydroxy2,2′bipyrrole5methanol synthase PigH	
SM24	1.89↓	0.01	4408		31		11		V3TRB4	Acetylcoenzyme A carboxylase carboxyl transferase subunit alpha	
SM25	1.82↓	0.05	8134		16.2		16		V3V2S3	Flagellar M-ring protein	
SM26	1.80↓	0.03	19030		37.2		7		V3TVM1	3-deoxymannooctulosonate-8-phosphatase	
SM25	1.77↓	0.01	4.06e + 6		49.5		7		V3TM10	Uncharacterized protein	
SM28	1.72↑	0.01	21656		21.2		8		V3T6F0	Uncharacterized protein	
SM29	1.70↑	0.00	128494		24.4		10		V3T1L7	Butyryl CoA dehydrogenase	
SM30	1.68↓	0.02	6038		9.9		11		V3TL00	Betaketoacylacyl carrier protein synthase I	
SM31	1.62↓	0.04	9036		18.1		12		Q5W262	Beta-ketoacyl synthase PigJ	
SM32	1.60↓	0.07	1058		38.6		5		V3TQE0	Uncharacterized protein	
SM33	1.59↓	0.06	110721		13.2		10		V3T592	Aspartate ammonia lyase	
SM34	1.58↑	0.02	207963		22.3		9		V3T1L7	Butyryl CoA dehydrogenase	
SM35	1.54↑	3.6E-04	16749		18.8		7		V3V2B2	Uncharacterized protein	
SM36	1.52↑	0.01	1676		34.9		9		V3TYA1	Uncharacterized protein	
SM37	1.52↓	0.02	140135		21.8		7		V3TRK2	Cysteine synthase	
SM38	1.52↓	0.00	102439		27		27		V3T1K6	Betaketoacylacylcarrierprotein synthase I, 6deoxyerythronolide B synthase	
SM39	1.50↓	0.04	16330		16.5		8		V3TMG3	Signal recognition particle receptor FtsY

Vanillic acid treatment down regulated the expression of surface layer protein (slaA) in *S. marcescens* by 4.68 fold. Protein surface layer (S-layers) present in Gram-positive and Gram-negative bacteria has been known to involve in cell stabilization (mechanical, thermal and osmotic)^33^, compartmentalisation, protection from host immunological defences (phagocytosis)^34^, trapping of ions and immobilization of proteins^35^. SlaA of *S. marcescens* is partially similar to the *Caulobacter crescentus* paracrystalline S-layer protein and is recognized and transported into extracellular medium by the Lip system^36^. Role of slaA in biofilm formation of *S. marcescens* has not been identified yet. However, S-layer has been shown to have a role in adherence and maintaining cell surface hydrophobicity (CSH) of certain Gram-positive and Gram-negative bacteria^37^ and is one of the determinants required for the biofilm formation. Hence, down-regulation of slaA by vanillic acid could modulate the CSH in *S. marcescens* which is expected to affect the biofilm formation. Furthermore, vanillic acid also down-regulated (−2.11 fold) the expression of flagellin and upregulated (+2.57 fold) the expression of the long polar fimbrial chaperone (LpfB). In *S. marcescens*, flagellin is synthesized by the *flhDC* operon and polymerized into flagella, whereas LpfB is involved in the organization of cell wall and pili. Disruption of *flhDC* operon in *S. marcescens* CH−1 has been shown to be defective in swarming motility^38^. Flagella and swarming motility required for the initial attachment of biofilm formation^39^. Hence, down-regulation of flagellin by vanillic acid could be responsible for the reduction in biofilm formation. In addition, flagellar M-ring protein was also down-regulated upon vanillic acid treatment. Aminohydrolases are involved in nucleotide and amino acid metabolism^40^. Proteomic analysis revealed a 2 fold down-regulation of the expression of amidohydrolase of *S. marcescens* upon vanillic acid treatment.

Expression of probable phospholipid-binding protein (MlaC) was found to be upregulated upon vanillic acid treatment. MlaC is involved in bacterial intermembrane phospholipid trafficking^41^ and *mlaC* mutant of *Escherichia coli* has been shown to have increased phospholipids content in the outer membrane^42^. Hence upregulation of MlaC by vanillic acid could alter the phospholipid content of *S. marcescens* outer membrane. Urocanate hydratase (HutU) is involved in histidine metabolism found to down-regulated upon vanillic acid treatment by 2.70 fold when compared to control. Biosynthesis of purines and pyrimidines depends on the availability of histidine and DNA synthesis is linked to e-DNA release, which is also required for the biofilm formation^43^. In addition, HutU has been reported to have biofilm formation of *Acinetobacter oleivorans* ^44^ and *Acinetobacter baumannii* ^43^. It is likely that down-regulation of HutU by vanillic acid could also be responsible for the reduction of biofilm formation in *S. marcescens*.

UniProtKB shows that uncharacterized protein A0A0U6CCF3 is 90% identical in terms of sequence similarity to the biofilm development protein (bsmB [A0A0N1UW53]) of *S. marcescens subsp. marcescens* Db11. In *S. marcescens*, bsmB has already been shown to regulate by AHL mediated QS and plays a crucial role in biofilm development^22^. Recently phytol has been reported to inhibit the biofilm formation in *S. marcescens* by targeting the expression of bsmB^45^. Hence, we hypothesized that down-regulation of uncharacterized protein A0A0U6CCF3 in *S. marcescens* CI by vanillic acid could be responsible for the biofilm inhibition.

Enzymes such as beta-ketoacyl synthase (PigJ) and 4-hydroxy- 2, 2′- bipyrrole-5-methanol synthase (PigH) involved in prodigiosin biosynthesis of *S. marcescens* ^15^ were found to be down-regulated upon vanillic acid treatment. These results further validate the prodigiosin inhibitory potential of vanillic acid. Furthermore, 3-deoxymannooctulosonate-8-phosphatase (KdsC) which is known to involve in the biosynthesis of lipopolysaccharide and bacterial outer membrane synthesis^46^ was found to be down-regulated upon vanillic acid treatment. Since components of outer membrane and LPS are immunogenic and required for biofilm formation in Gram-negative bacterial pathogens^46,47^, down regulation of KdsC by vanillic acid could also be responsible for the reduction in the biofilm of *S. marcescens*.

Proteomic analysis of vanillic acid treated *S. marcescens* revealed the upregulation of multiple stress resistance protein (bhsA), which is predicted to be involved in stress response mechanism. BhsA of *S. marcescens* is 52.1% similar to putative outer membrane protein ycfR (bhsA) of *E. coli* in terms of the sequence. In *E. coli*, deletion ycfR (bhsA) has been shown to increase the biofilm, aggregation and CSH^48^. Upregulation of bhsA upon vanillic acid treatment could affect the biofilm formation in *S. marcescens*. In addition, vanillic acid treatment was found to upregulate the expression of 60 kDa chaperonin (groL) protein. GroL prevents the misfolding and helps in refolding/proper folding of proteins synthesized under stress condition^49^. Beta-ketoacyl-acyl carrier protein synthases are involved in fatty acid biosynthesis in most of the bacteria. Hence they have been identified as one of the promising targets for broad-spectrum antibacterial agents^50^. Down-regulation of beta-ketoacyl-acyl carrier protein synthase I, acetyl-coenzyme A carboxylase carboxyl transferase subunit alpha and beta-ketoacyl-acyl-carrier-protein synthase I, 6-deoxyerythronolide B synthase suggested the ability of vanillic acid to interfere the fatty acid biosynthesis of *S. marcescens*. Furthermore, the expression of 3-oxoacyl-[acyl-carrier-protein] reductase (fabG) of *S. marcescens* was down regulated by vanillic acid. FabG has already been reported to regulate the virulence of *Pseudomonas syringae* pv. tabaci through AHL and fatty acid biosynthesis^51^. Whereas, in *P. aeruginosa* fabG determines the acyl chain length of 3-oxo-C_12_-HSL^52^. Role of fabG in QS and biofilm formation of *S. marcescens* has not been established yet. To get a preliminary information global sequence alignment analysis was carried out using EMBOSS Needle and the results revealed that the fabG of *S. marcescens* shares 64.8 and 78.1% identity and similarity, respectively to fabG of *P. syringae* pv. tabaci (A0A0W0PM71) and 63.2 and 75.3% identity and similarity, respectively to fabG (O54438) of *P. aeruginosa*. Hence it is hypothesized that down-regulation of fabG by vanillic acid could have a negative effect on QS-regulated virulence factors production in *S. marcescens*. In addition to inhibition of fatty acid biosynthesis pathway, amino acid (arginine, proline, cysteine and methionine) and butanoate metabolism have been predicted to inhibit the biofilm formation^53^. In the present study also down-regulation (1.5 fold) of cysteine synthase was noted upon vanillic acid treatment. Furthermore, down-regulation in the expression of aspartate ammonia lyase (aspA) was observed upon vanillic acid treatment. AspA has been reported to involve in the conversion of L-aspartate into fumarate and ammonia^53^. AspA mediated ammonia generation has already been reported to increase the intracellular polyamines synthesis, which in turn alters the membrane permeability, increases resistance to antibiotics and oxidative stress^54^. In contrast, the expression of butyryl-CoA dehydrogenase of *S. marcescens* was upregulated upon vanillic acid treatment. These results revealed the ability of vanillic acid to modulate the expression of proteins involved in the fatty acid and amino acid biosynthesis in *S. marcescens* CI.

**Figure 7:**
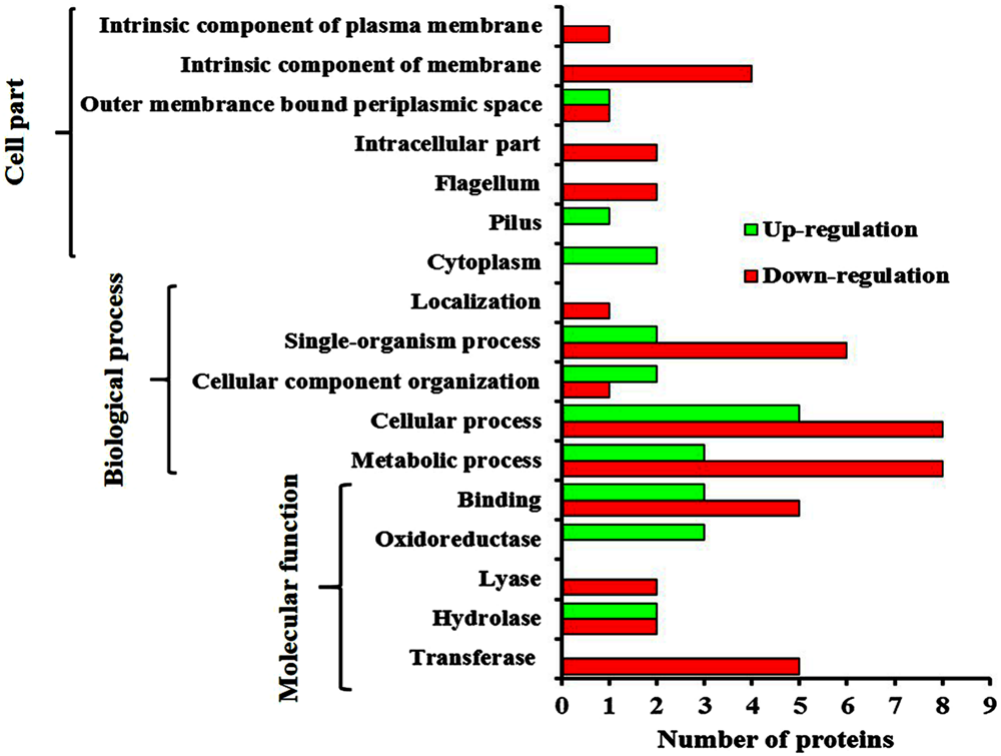
MR-VP test results depicting the effect of vanillic acid treatment on acid (**a**) and butanediol (**b**) production in *S. marcescens* CI. Effect of different concentration of vanillic acid treatment on the CSH (**c**) and the growth of *S. marcescens* CI in the presence of 0.5 and 1 M NaCl (**d**). Error bars represent standard deviations from the mean (n = 3). *(p < 0.05) indicates the statistical value of vanillic acid treated compared to control. Error bars represent standard deviations from the mean (n = 3 [biological triplicates in experimental duplicates]). Statistical significance was analyzed using one way ANOVA-Duncan’s *post-hoc* test. ^a, b^and ^c^ indicate the significant difference p < 0.05, p < 0.01 and p < 0.005, respectively.

Mutants of *Serratia plymuthica* RVH1 lacking *splI* (AHL synthase), has been shown to modulate the mixed-acid and butanediol fermentation. Briefly, mutation in *splI* gene resulted in enhanced and decreased acid and butanediol production respectively, in *S. plymuthica* ^55^. Interestingly in the present study vanillic acid treatment resulted in the upregulation of 2, 3-butanediol dehydrogenase (budC) of *S. marcescens*, which is under the control of QS. For further confirmation, Methyl Red Voges Proskauer (MR-VP) test was carried out in the present investigation and the results revealed that, vanillic acid treatment enhances the acid production and decreases the butanediol production. Though, 2, 3-butanediol dehydrogenase (budC) is upregulated upon vanillic acid treatment, the production of butanediol is reduced when compared to control (Fig. 7a and b). Butanediol production in *S. plymuthica* RVH1 and *S. marcescens* MG1 is regulated by AHL mediated QS and the growth of *S. marcescens* was not altered in the presence of vanillic acid. Hence reduction of butanediol production in *S. marcescens* upon vanillic acid treatment suggests its ability to modulate the AHL synthesis.

In addition, vanillic acid treatment down regulated the expression of signal recognition particle receptor (FtsY) protein by 1.5 fold. FtsY is actively involved in the translocation/insertion of nascent membrane proteins into the cytoplasmic membrane^56^. Furthermore, molecular chaperone OsmY was found to be down regulated upon vanillic acid treatment in *S. marcescens*. EMBOSS Needle global sequence alignment analysis revealed that OsmY is 91.7% identical and 95.6% similar in terms of sequence with transport-associated protein (A8G9G9) of *Serratia proteamaculans* and which is shown to interact with the lipid A 1-diphosphate synthase (lpxT) and outer membrane protein assembly factor (BamA) involved in LPS biosynthesis and assembly of outer membrane proteins, respectively [interaction can be found in STRING database]^57^. Down-regulation of FtsY and OsmY suggests the possible involvement of vanillic acid to modulate the transport of membrane proteins.

Vanillic acid treatment was found to upregulate the expression of aldehyde dehydrogenase B (+4.74 fold) in *S. marcescens*. Recently, aromatic aldehyde dehydrogenases from *Sphingobium* sp. strain SYK-6 have been reported to catalyze the conversion of vanillin to syringaldehyde^58^. Hence, upregulation of aldehyde dehydrogenase B could be the possible mechanism responsible for the survival of *S. marcescens* in the presence of vanillic acid. Oxidoreductase has been reported to regulate the biofilm formation and virulence in *Salmonella enterica* serovar Typhimurium^59^ and *Burkholderia cenocepacia* ^60^. In the present study, vanillic acid treatment was found to down-regulate the expression of putative oxidoreductase by 2.7 fold. In addition, vanillic acid treatment was found to modulate the expression of ferrous iron transporter B (+5.77 fold), delta-aminolevulinic acid dehydratase (+2.40 fold) and several uncharacterized proteins (Table 1).

Role of slaA in biofilm formation and osmotic stress resistance in *S. marcescens* has not been studied yet. However, surface layer proteins of *Lactobacillus acidophilus* ATCC 4356 have been shown to protect the cells form NaCl induced osmotic stress^61^ and are responsible for adherence and cell surface hydrophobicity (CSH)^37^. To this end, CSH assay was carried out and the results revealed the concentration dependent reduction in CSH of *S. marcescens* upon vanillic acid treatment (Fig. 7c). Reduction in the CSH hinted the possible role of slaA in the CSH of *S. marcescens*. Furthermore, in the present study, we have also evaluated the effect of vanillic acid treatment on the osmotic stress resistance of *S. marcescens* and the results revealed that vanillic acid treated cells are unable to grow normally in the presence of 0.5 and 1 M NaCl when compared to control. These results suggest the ability of vanillic acid to modulate the osmotic stress resistance in *S. marcescens* by down regulating the expression of slaA protein. Further analyses are required to unveil the role of slaA in biofilm formation and virulence of *S. marcescens*.

## Summary

In conclusion, vanillic acid has been identified as an active principle responsible for QSI and antibiofilm potential of kiwifruit (*A. deliciosa*) against *S. marcescens*. Virulence assays revealed the concentration dependent inhibitory activity over QS regulated biofilm, protease, prodigiosin and lipase production and swarming motility of *S. marcescens* CI and *S. marcescens* ATCC. In addition, vanillic acid also inhibited the protease and lipase production and biofilm formation in non-pigmented *S. marcescens* MG1. In addition, light and CLSM microscopic analyses further confirmed the antibiofilm activity of vanillic acid against *S. marcescens* CI, *S. marcescens* ATCC and *S. marcescens* MG1 used in this study. Furthermore, FTIR analysis of EPS extracted from control and vanillic acid treated *S. marcescens* CI showed the reduction in the nucleic acid, amide I and II proteins and fatty acid content. *In vivo* assays confirms the ability of vanillic acid to rescue *C. elegans* form the *S. marcescens* CI, *S. marcescens* ATCC and *S. marcescens* MG1 infection by inhibiting the QS regulated virulence and biofilm formation. Mass spectrometric identification of differentially expressed proteins and gene ontology analyses revealed the ability of vanillic acid modulates to the proteins involved in S-layers, flagellin, amino acid and fatty acid production in *S. marcescens* CI. The present study suggests that vanillic acid with its non-toxic nature and QSI potential can serve as a choice to potentiate the treatment strategy to overcome the QS- and biofilm-mediated infections caused by *S. marcescens*.

## Materials and Methods

### Ethical statement

Sheep blood was collected from the Karaikudi municipality modern slaughter house, Karaikudi and used to evaluate the effect of vanillic acid treatment on the hemolysin production in *S. marcescens*. Normally sheep blood is discarded in the butchery and hence no specific ethical permission was needed.

### Collection of plant material and preparation of *A. deliciosa* pulp extract (ADPE)

*A. deliciosa* (Kiwifruit Zespri International Limited, New Zealand) was collected from the local market and the pulp was ground and centrifuged to collect the *A. deliciosa* pulp extract (ADPE). The ADPE was filter sterilized using 0.2 µm on the membrane filter (Millipore Corp., USA).

### Bacterial strain and growth conditions

*S. marcescens* Clinical isolate used in this study is a clinical strain isolated from a urine sample, identified by 16S rRNA gene sequencing (GenBank Accession Number: FJ584421). In addition, *S. marcescens* ATCC 14756 and *S. marcescens* MG1 was also used to evaluate the anti-QS and antibiofilm activity of vanillic acid.

To determine the effect of ADPE on QS regulated extracellular virulence factors, LB medium (1% tryptone, 1% sodium chloride and 0.5% yeast extract) supplemented with and without (10–20% v/v, in increments of 5%) filter sterilized ADPE was inoculated with 1% (v/v) of the overnight culture of *S. marcescens* (OD_600nm_ = 0.5) and incubated at 30 °C for 18 h at 120 rpm. After incubation, the cell density of control and treated *S. marcescens* CI was measured at 600 nm followed by centrifugation at 12,000 rpm for 15 min at 4 °C to collect the cell free culture supernatant (CFCS). CFCSs were stored at −20 °C for protease, lipase and hemolysin assay. The cell pellet was used for prodigiosin assay. Experiments are performed in biological triplicates in experimental duplicates.

### Measurement of prodigiosin

For extraction of prodigiosin, cell pellets were resuspended in 1 ml acidified ethanol (4% (v/v) of 1 M Hydrochloric acid (HCl) in ethanol) and vortexed vigorously. Acidified ethanol containing solubilized prodigiosin and cell debris was subjected to centrifugation at 10,000 rpm for 10 min and the optical density of the supernatant was measured at 534 nm. The percentage of prodigiosin inhibition was calculated using the formula^17^:

$${\rm{ \% }}\,{\rm{o}}{\rm{f}}\,{\rm{i}}{\rm{n}}{\rm{h}}{\rm{i}}{\rm{b}}{\rm{i}}{\rm{t}}{\rm{i}}{\rm{o}}{\rm{n}}=({\rm{c}}{\rm{o}}{\rm{n}}{\rm{t}}{\rm{r}}{\rm{o}}{\rm{l}}\,{{\rm{O}}{\rm{D}}}_{534{\rm{n}}{\rm{m}}}-{\rm{t}}{\rm{e}}{\rm{s}}{\rm{t}}\,O{D}_{534{\rm{n}}{\rm{m}}}/\text{control}\,{{\rm{O}}{\rm{D}}}_{534{\rm{n}}{\rm{m}}})\times 100.$$

### Total protease assay

The casein-degrading proteolytic activity was assessed by incubating each 100 µL reaction mix containing 2 mg/mL of azocaesin in 0.05 M Tris-hydrochloride, 0.5 mM CaCl_2_ (pH 7.5) with 100 µl of control and ADPE treated *S. marcescens* CFCS at 37 °C for 15 min. A 500 µl of 10% trichloroacetic acid was added to each reaction tube to terminate the reaction, followed by incubation at −20 °C for 10 min and the tubes were centrifuged at 12000 rpm for 20 min. Finally, the absorbance of the supernatant was measured at 400 nm using Multi-Mode Microplate Reader (SpectraMax M3, US)^12^.

### Lipase assay

A 100 µl of *S. marcescens* culture supernatant (control and ADPE treated) was incubated with 900 µl of reaction mixture containing 1 volume 0.3% (w/v) p-nitro phenyl palmitate in isopropanol and 9 volumes of 50 mM Na_2_PO_4_ buffer (pH 8.0) containing 0.2% (w/v) sodium deoxycholate and 0.1% (w/v) gummi arabicum for 1 h at room temperature in dark. An equal volume of 1 M Na_2_CO_3_ was added to each tube to terminate the lipolytic activity and then centrifuged at 12,000 rpm for 10 min at room temperature. The absorbance of the supernatant was measured at 410 nm^62^.

### Hemolysin assay

Hemolysin production was measured in accordance with the method described earlier^18^. Equal volume of 2% sheep red blood cells (RBCs) in phosphate buffered saline (pH 7.4) was mixed with control and ADPE treated *S. marcescens* CFCS at 37 °C for 2 h. After incubation, the reaction mixture was centrifuged at 8,000 rpm for 5 min and the absorbance of the supernatant was measured at 530 nm^63^.

### Biofilm inhibition assay

Anti-biofilm potential of ADPE extract was determined using the method adopted by our group previously^64^. The biofilm of *S. marcescens* was allowed to grow in LB medium in the presence (10–20% v/v, in increments of 5%) and absence of ADPE in 24-well polystyrene plate at 30 °C for 24 h. After incubation, the wells were washed with distilled water to remove the planktonic cells. The sessile cells were stained with 0.4% crystal violet stain (CV) (w/v) for 5 minutes followed by washing off the unstained dye using distilled water. Finally, 1 mL of absolute ethanol was added to each well to dissolve the CV present in the biofilm cells. The absorbance (OD_570nm_) was determined and percentage of biofilm inhibition was calculated using the formula:

$$ \% \,{\rm{of}}\,{\rm{inhibition}}=({\rm{control}}\,{{\rm{OD}}}_{570{\rm{nm}}}-{\rm{test}}\,{{\rm{OD}}}_{570{\rm{nm}}}/\text{control}\,{{\rm{OD}}}_{570{\rm{nm}}})\times 100.$$

### Successive extraction of *A. deliciosa* pulp extract

Freeze-dried *A. deliciosa* pulp was ground into fine powder using a blender and extracted with 70% ethanol for 3 h at boiling point and filtered through Whatman filter paper No. 2. The filtered *A. deliciosa* pulp ethanolic extract (ADPEE) was evaporated under vacuum at 55 °C for the complete removal of water and ethanol. Then the ADPEE was dissolved in distilled water and extracted successively with hexane (ADPEE-H), chloroform (ADPEE-C), ethyl acetate (ADPEE-E), and n-butanol (ADPEE-B), finally leaving an aqueous fraction of ethanol extract (ACPEE-W)^65^. Above mentioned solvent extracts were dried under vacuum and assayed for their QSI activity against *S. marcescens* as mentioned previously.

### GC-MS analysis of ADPEE-C

ADPEE-C extract exhibited concentration dependent inhibition of QS regulated virulence factors of *S. marcescens*. Thus ADPEE-C was analysed by SHIMADZU GCMS-138 QP2010 plus, containing Rxi®-5ms gas chromatograph column (ID 0.25 mm thickness 0.25 139 µm), coupled to a mass spectrometer mass detector (with 5% diphenyl and 95% 140 dimethylpolysiloxane) using standard GC-MS parameters. The constituents of ADPEE-C were identified by processing of the raw GC-MS data with the NIST software (National Institute of Standards and Technology, Gaithersburg, USA).

### Effect of pure vanillic acid on the QS regulated virulence factors production of *S. marcescens* strains

Pure vanillic acid (Sigma, USA) was dissolved in ethanol (100 mg/mL). *S. marcescens* CI, *S. marcescens* ATCC and *S. marcescens* MG1 were grown in the presence and absence of 62.5, 125, 250 and 500 µg/mL vanillic acid and incubated at 30 °C for 18 h at 120 rpm. After incubation growth, prodigiosin, lipase, protease, hemolysin and biofilm formation were measured as mentioned previously.

### CLSM analysis of biofilm

For CLSM analysis, biofilm slides were prepared by growing *S. marcescens* CI, *S. marcescens* ATCC and *S. marcescens* MG1 in the absence and the presence of 250 and 500 µg/mL of vanillic acid for 24 h at 30 °C. The biofilms formed on the glass slides were stained with 0.1% acridine orange for 1 min, washed with distilled water, air dried and visualized under CLSM (Carl Zeiss, Germany) at 20× magnification. CLSM images (N = 3) were obtained from the triplicate of control and treated biofilm and processed using Zen 2009 image software^64^.

### Swarming motility assay

The effect of vanillic acid on the swarming motility was determined by placing 2 µL of overnight cultures of *S. marcescens* in the centre of swarm agar plates (peptone (5 g/L), glycerol (1% (v/v)) and agar (0.75%)). The plates were incubated at 25 °C for 20 h. The activity was determined by the decrease in the swarmed area radius^5^.

### Extraction of EPS and FTIR analysis

EPS extraction was carried out in accordance with the previously published methods^66^. Briefly, each 100 mL of *S. marcescens* CI control and vanillic acid treated cultures were centrifuged at 7, 000 rpm for 15 min at 4 °C to collect the cells. Cell free culture supernatants were stored at −20 °C for cell free EPS extraction. The collected cell pellets were washed with wash buffer (10 mM Tris/HCl pH 8.0, 10 mM EDTA) and resuspended in 100 mL of isotonic extraction buffer (10 mM Tris/HCl pH 8.0, 10 mM EDTA, 0.5 mM NaCl) and incubated at 4 °C for 12 h. After incubation, isotonic buffer containing cells were vortexed for 5 min and centrifuged at 5000 rpm for 15 min to collect the supernatant containing cell bound EPS. Then, both CFCS containing cell free EPS and isotonic buffer containing cell bound EPS were pooled and mixed with 3 volumes of ice-cold ethanol and kept at −20 °C for 18 h for the precipitation of EPS. Precipitated EPS was collected by centrifugation at 12, 000 rpm for 30 min at 4 °C and dried under vacuum^62^. For FTIR analysis, each 2 mg of EPS from control and treated were mixed with 100 mg of potassium bromide (KBr) to prepare KBr-EPS pellet and infrared spectra were collected in the range of 400 to 4000 cm^−1^ using FTIR spectrometer (Bruker Tensor 27) and values were plotted as intensity versus wave number.

### Caenorhabditis elegans maintenance

*C. elegans* wild type N2 worms were obtained from Caenorhabditis Genetic Centre (CGC) Minnesota, USA and maintained in Nematode Growth Medium (NGM) containing *E. coli* OP50 as a food source. Synchronised L4 stage animals were used for all the experimental. To obtain synchronised L4 stage worms, adult stage animals were bleached with 1:1 ratio of commercial bleach and 1 M KOH^67^.

### Toxicity and liquid killing assay

To check the toxicity of vanillin, 15 numbers of synchronised L4 stage *C. elegans* were transferred to M9 buffer (500 µL) containing *E. coli* OP50 (~1000 CFU/mL), presence and absence of vanillin 250 µg/mL. Ethanol was used as a vehicle in all the experiments. To evaluate the anti-QS and antibiofilm activity of vanillic acid, 15 numbers of synchronised L4 stage *C. elegans* were transferred to 24 well microtitre plate containing 400 µl of M9 buffer + 100 µL of *S. marcescens* CI and *S. marcescens* MG1 culture with 0.3 OD at 600 nm and supplemented with and without vanillic acid 250 µg/mL. In the case of *S. marcescens* ATCC 14756, 500 µg/mL of vanillic acid was used. The assay was monitored continuously for every 6hrs interval until the complete death of *C. elegans* in control. The animals did not show any response to touch by the external stimuli like platinum worm picking loop then it was considered as dead^67^.

### Microscopic observation

For microscopic observation, *C. elegans* were exposed to *S. marcescens* CI, *S. marcescens* ATCC and *S. marcescens* MG1, with and without vanillic acid for 24hrs. Then control and treated *C. elegans* were washed with M9 buffer thoroughly and placed on a microscopic slide with 1 mM Sodium azide and visualized under bright field microscope^68^.

### Colony forming unit assay (CFU)

To access the intestinal colonization of *S. marcescens* CI, *S. marcescens* ATCC and *S. marcescens* MG1 in *C. elegans* CFU was performed. Briefly synchronised L4 stage *C. elegans* were exposed to *S. marcescens* CI, S. marcescens ATCC and *S. marcescens* MG1, with and without vanillin 250 µg/mL for 24hrs. After 24hrs, 20 numbers of exposed worms were washed thoroughly with M9 buffer containing 1 mM sodium azide to reduce the discharge of bacteria from the worm intestine and finally crushed/ground with 400 mg of 1.0 mm of silicon carbide and vortexed for 20 minutes. The final suspension was serially diluted and plated onto specific medium^68^.

### Extraction of cellular proteins

For extraction of intracellular proteins, *S. marcescens* was grown in the absence and presence of 250 µg/mL of vanillic acid for 18 h at 30 °C with 120 rpm in conical flasks in biological triplicates and cells were harvested by centrifugation at 8000 rpm for 12 min at 4 °C and the resulting pellets were washed twice with 50 mM tris-HCl (pH 8.0). Cell pellets were resuspended in 50 mM tris-HCl (pH 8.0) supplemented with 1% of protease inhibitor cocktail and sonicated using Ultra-sonicator (Sonics VCX 750, USA) with the parameters of 35 KHz, 750 W and 35% amplitude for 5 min with pulse time of 10 sec (for both on and off cycles) followed by centrifugation at 13,000 rpm for 30 min at 4 °C to remove the cell debris. Supernatants containing cellular proteins were purified using phenol extraction and precipitated by acetone. Precipitated proteins were washed thrice with ice-cold acetone to wash off the remaining phenol residues and air dried. Finally, the air-dried protein pellets were dissolved in urea thiourea sample buffer (7 M urea, 2 M thiourea, 4% 3-[(3-cholamidopropyl)-dimethylammonio]-1-propanesulfonate [CHAPS], 15 mM dithiothreitol [DTT] and 0.5% of immobilised pH gradient [IPG] buffer [pH 3–10]) and centrifuged at 13,000 rpm for 20 min at 4 °C and the concentration of proteins present in the samples were quantified by Bradford assay kit (BioRad). Each 450 µg of cellular proteins form control and vanillic acid treated *S. marcescens* were used for isoelectric focusing (IEF) in biological triplicates^69^.

### Two-dimensional gel electrophoresis (2DGE) and image analysis

Protein samples (each 400 µg) from control and vanillic acid treated *S. marcescens* were diluted with urea thiourea rehydration buffer (7 M urea, 2 M thiourea and 2% [CHAPS]) supplemented with 12.5 mg/mL destreak reagent and 0.5% IPG buffer [pH 3–10] to a final volume of 350 µl. Immobiline DryStrip gel strips (18 cm, non-linear, pH 3–10) were rehydrated with thiourea rehydration buffer containing cellular proteins for 12 h at 20 °C and the rehydrated strips were iso-electrically focused in IPGphor 3 system using standard parameters. After IEF, IPG strips were subjected to reduction and alkylation with DTT and iodoacetamide (IAA), respectively. Then the strips were placed on 25 cm × 22 cm × 1 mm 10–15% gradient sodium dodecyl sulphate-polyacrylamide gels (SDS-PAGE), sealed with 0.3% agarose solution containing a trace amount of tracking dye Bromophenol blue and electrophoresis was carried out at 100 V for 1 h and 150 V until the dye front had reached the bottom of the SDS-PAGE gel in Ettan DALT six apparatus (GE Healthcare). After electrophoresis, the gels incubated in fixative solution (40% methanol, 10% glacial acetic acid (GAA) and 50% MilliQ H_2_O) for 3 h. Protein spots were visualized using mass spectrometry compatible colloidal coomassie brilliant blue (CBB) G-250 staining for 12 h and destained with MilliQ H_2_O until the background becomes clear. Then, image acquisition was done with Image Scanner III (GE Healthcare) and analysed by ImageMaster 2D Platinum 7.0 software (GE Healthcare)^69^.

### Protein spots excision and *in-gel* trypsin digestion

The protein spots with 1.5 fold differential regulation (up/down) were excised from the gels and the excised gel plugs were destained with 50% acetonitrile (ACN) containing 25 mM ammonium bicarbonate (NH_4_HCO_3_) until the complete removal of CBB. Destained gel plugs were dehydrated in ACN, vacuum dried and rehydrated with 5 μL of digestion buffer (10 mM NH_4_HCO_3_ in 10% ACN) containing 400 ng of sequencing grade trypsin (Sigma Aldrich) on ice for 30 min. After rehydration with digestion buffer, gel plugs were flooded with 25 μL of overlay solution (40 mM NH_4_HCO_3_ in 10% ACN) and incubated at 37 °C for 16 h. After trypsin digestion, peptides were extracted with peptide extraction solution (0.1% trifluoroacetic acid (TFA) in 60% ACN) by sonication followed by complete dehydration of gel plugs with ACN. Collected peptides were pooled and vacuum dried for 90 min and stored at 4 °C^70^. For nano LC-MS/MS and MALDI-TOF/TOF analyses, vacuum dried peptides were purified using C_18_ zip tips (Merck Millipore) as per the manufacturer’s instructions.

### Identification of differentially expressed proteins by nano LC-MS/MS and MALDI-TOF/TOF analyses

Protein spots with ≥2 fold differential regulation were analysed using nano reverse phase-LC (Thermo Scientific, USA) coupled with an Orbitrap Elite Mass spectrometer (Thermo Scientific, USA) using standard parameters^71^. The MS data acquisition was done in positive ion mode (m/z 350–4000 Da) using Xcalibur software (Version 2.2.SP1.48, Thermo Scientific, USA). Protein identification was done with Proteome Discoverer software v.1.4 (Thermo Scientific) using the following parameters: 2 missed cleavages, 10 ppm and 0.5 Da as precursor mass tolerance and fragment mass tolerance, respectively. In addition, cystine carbamidomethylation as fixed modification, methionine oxidation, N-terminal acetylation and phosphorylation (S, T, Y) as variable modification.

Protein spots with 1.5 to 1.9 fold differential regulation were analysed using MALDI-TOF/TOF analysis. Prior to analysis, internal calibration was carried out using TOF-Mix™ (LaserBio Labs, France). For MALDI-TOF/TOF analysis, 1 µL of purified peptides were mixed with 1 µL of the alpha-cyano-4-hydroxy cinnamic acid matrix (10 mg/mL) on MALDI target plate. Mass spectra were acquired in positive reflectron mode and the monoisotopic peak list (m/z range of 700–4000 Da) was used for protein identification using MS-Fit (http://prospector.ucsf.edu) online software using standard parameters^69^.

### MR-VP test

*S. marcescens* was allowed to grow in MR-VP broth supplemented with and without 125 and 250 µg/mL of vanillic acid for 24 h. After incubation, to detect the acid production, methyl red indicator dye was added and the changes in the colour was visually observed and photographed. To detect the butanediol formation Barritt’s reagent A and B were added to the culture, mixed well and formation of red coloration was visually observed and photographed.

### Osmotic sensitivity assay

To analyse the effect of vanillic acid treatment on the osmotic sensitivity, *S. marcescens* was allowed to grow in LB medium supplemented with 0.5 and 1 M NaCl in the presence and absence of 125 and 250 µg/mL of vanillic acid at 30 °C and cell density was measured periodically for 24 h with 2 h interval^61^.

### Statistical analysis

All the experiments were performed independently in triplicate to confirm reproducibility. Data were analyzed by one-way analysis of variance (ANOVA) followed by Duncan’s *post-hoc* test with a significant P value of <0.05 using the SPSS (Chicago, IL, USA) statistical software package.

## Electronic supplementary material

Supplementary Information
