# Supplementary material for: Vanillic acid from Actinidia deliciosa impedes virulence in Serratia marcescens by affecting S-layer, flagellin and fatty acid biosynthesis proteins
Source: Sci Rep. 2017 Nov 27;7:16328. doi: 10.1038/s41598-017-16507-x (PMC5703977; doi:10.1038/s41598-017-16507-x)

Vanillic acid from *Actinidia deliciosa* impedes virulence in *Serratia marcescens* by affecting S-layer, flagellin and fatty acid biosynthesis proteins

Sivasamy Sethupathy^a^, Sivagnanam Ananthi^b^, Anthonymuthu Selvaraj^a^, Balakrishnan Shanmuganathan^a^, Loganathan Vigneshwari^a^, Krishnaswamy Balamurugan^a^, Sundarasamy Mahalingam^b^, Shunmugiah Karutha Pandian^a^

^a^Department of Biotechnology, Alagappa University, Science Campus, Karaikudi 630 003, Tamil Nadu, India
^b^Laboratory of Molecular Virology and Cell Biology, Department of Biotechnology, Indian Institute of Technology Madras, Chennai 600 036, Tamil Nadu, India

*Correspondence and requests for materials should be addressed to SKP. (email:sk_pandian@rediffmail.com)

**Supplementary Figure 1.** Total ion chromatogram of ADPEE-C.


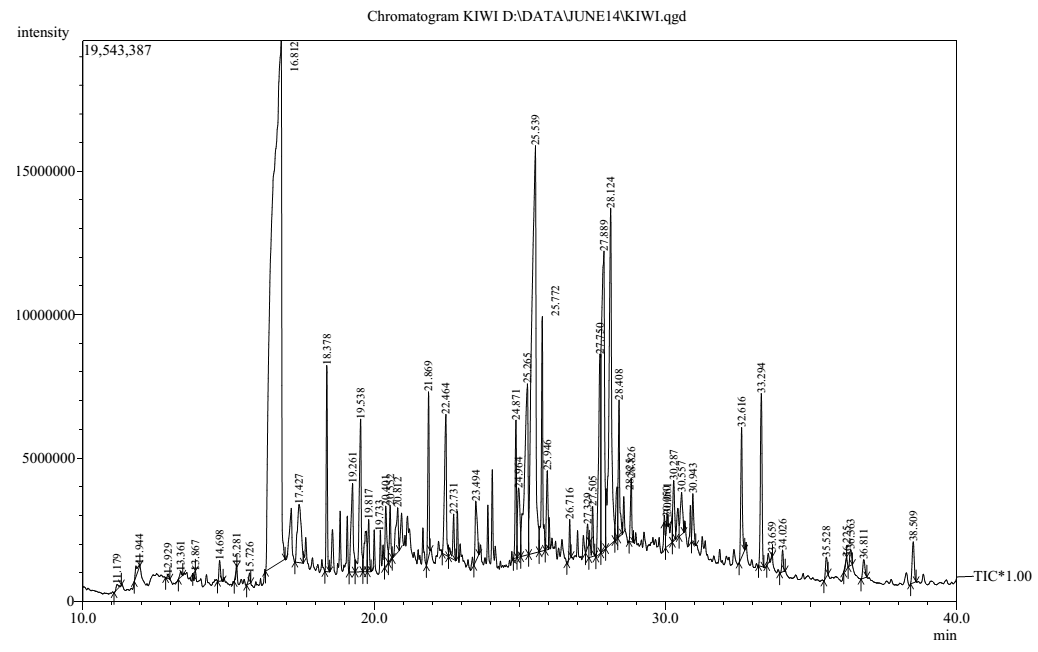


**Supplementary table 1.** Major constituents of ADPEE-C extract identified by GC-MS.

**
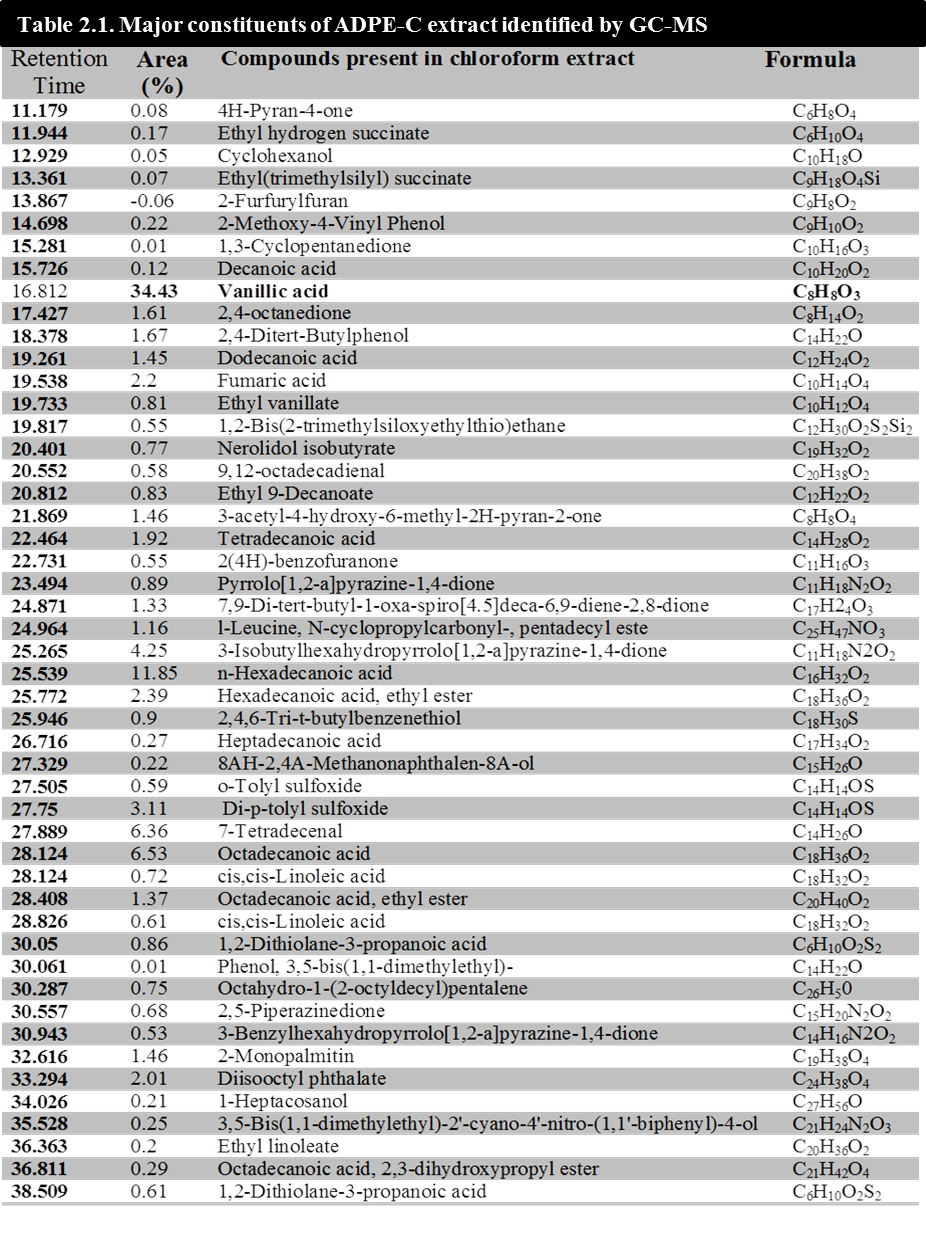
**

**Supplementary Figure 2.** Analysis of intracellular protein extract of *S. marcescens* grown in the absence and presence of 250 µg/mL of vanillic acid using 2-D gel electrophoresis. Each 450 µg of protein extract from control and treated cells were subjected to isoelectric focusing and resolved based on molecular weight in 10-15% gradient SDS-PAGE and protein spots were stained with MS compatible colloidal CBB.


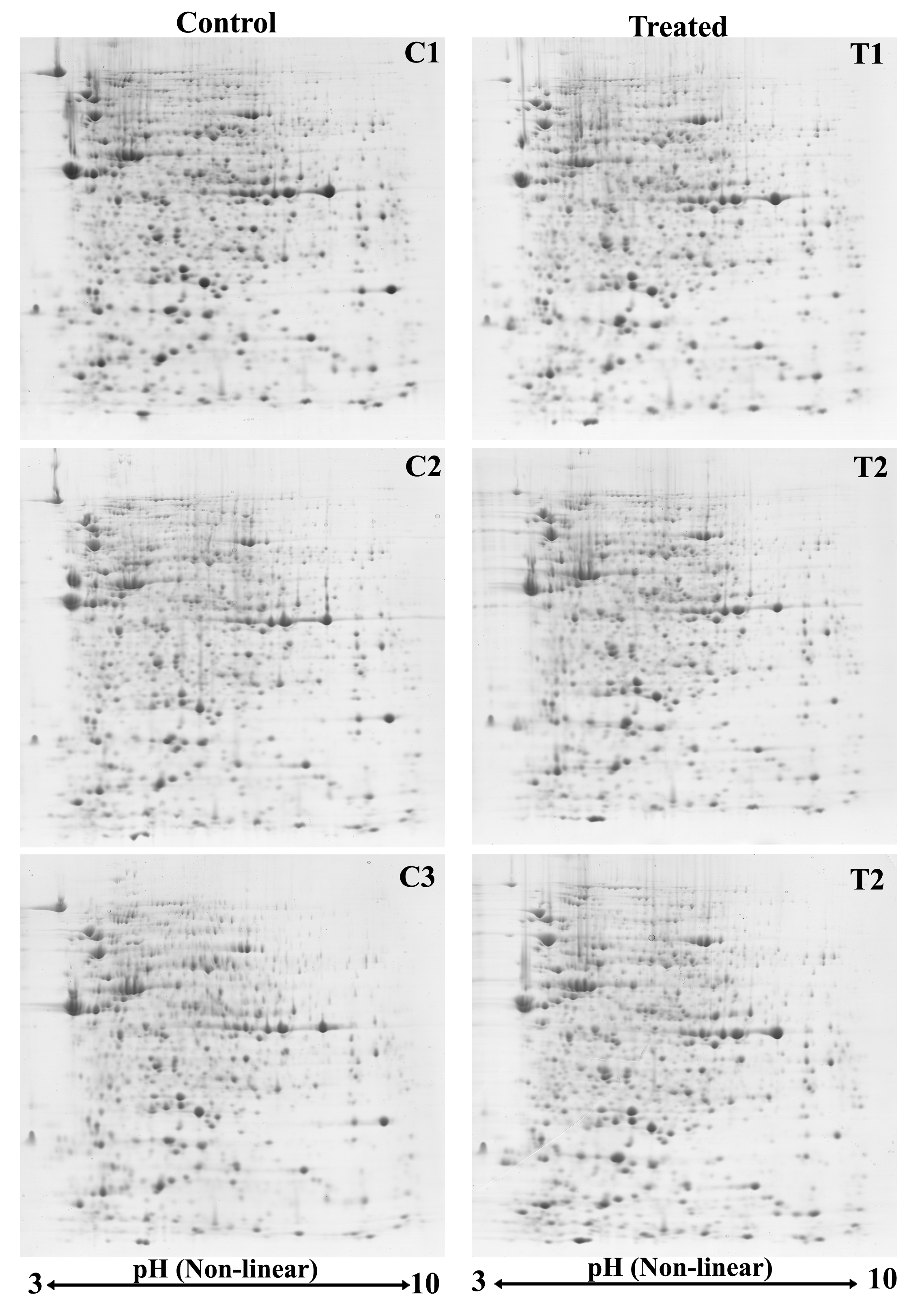

Supplement: Supplementary file 1 — Supplementary Information [file 41598_2017_16507_MOESM1_ESM.doc]
